# Electrically driven single-photon emission from an isolated single molecule

**DOI:** 10.1038/s41467-017-00681-7

**Published:** 2017-09-18

**Authors:** Li Zhang, Yun-Jie Yu, Liu-Guo Chen, Yang Luo, Ben Yang, Fan-Fang Kong, Gong Chen, Yang Zhang, Qiang Zhang, Yi Luo, Jin-Long Yang, Zhen-Chao Dong, J. G. Hou

**Affiliations:** Hefei National Laboratory for Physical Sciences at the Microscale and Synergetic Innovation Center of Quantum Information & Quantum Physics, University of Science and Technology of China, Hefei, Anhui 230026 China

## Abstract

Electrically driven molecular light emitters are considered to be one of the promising candidates as single-photon sources. However, it is yet to be demonstrated that electrically driven single-photon emission can indeed be generated from an isolated single molecule notwithstanding fluorescence quenching and technical challenges. Here, we report such electrically driven single-photon emission from a well-defined single molecule located inside a precisely controlled nanocavity in a scanning tunneling microscope. The effective quenching suppression and nanocavity plasmonic enhancement allow us to achieve intense and stable single-molecule electroluminescence. Second-order photon correlation measurements reveal an evident photon antibunching dip with the single-photon purity down to *g* ^(2)^(0) = 0.09, unambiguously confirming the single-photon emission nature of the single-molecule electroluminescence. Furthermore, we demonstrate an ultrahigh-density array of identical single-photon emitters.

## Abstract (web summary)

Molecular emitters offer a promising solution for single-photon generation. Here, by exploiting electronic decoupling by an ultrathin dielectric spacer and emission enhancement by a resonant plasmonic nanocavity, the authors demonstrate electrically driven single-photon emission from a single molecule.

## Introduction

Single-photon sources lie in the heart of quantum information technology^1, 2^, from quantum cryptography^3, 4^ to quantum computing^5, 6^. High-density single-photon emitters with well-defined source arrays hold the key for the construction of on-chip quantum devices^7^ and quantum networking^8^. Various single-emitter quantum systems, such as semiconductor quantum dots^9–13^, atoms^14–16^, color centers^17–19^, and molecules^20–25^, have been investigated as potential single-photon sources. Among them, molecular emitters have attracted extensive attention because of their various merits such as sharp emission, a wide range of tunable photon energies, identical emission behavior, and high stability^22, 26^. Elegant progress has also been made on electrically driven molecule-based single-photon emitters by using dispersed molecules in organic light-emitting diode devices^24^. The single-photon measurements there were performed by selecting a random emitter among many others in a stochastically dispersed sample, making it difficult to control the emitter environment and to eliminate the background disturbance. By contrast, scanning tunneling microscope (STM)-induced luminescence (STML), a technique capable of going beyond atomic resolution imaging and manipulation, can use highly localized tunneling electrons to excite single-molecule electroluminescence^27–34^, which is expected to be a single-photon source. Indeed, efforts have been made to probe the photon correlation for light generated in the tunnel junction by combining STML with the Hanbury Brown and Twiss (HBT) interferometry^35–38^. Nevertheless, available reports are very limited due to strong quenching effect near metals, amounting to mainly the observation of a photon bunching phenomenon for molecules in solution^37^ and an antibunching behavior associated with the single-photon emission from local structural defects related to a small number of C_60_ molecules in the C_60_ thin films^38^. Consequently, the goal to achieve electrically driven single-photon emission from a well-defined single molecule remains to be reached.

In this work, by using a combined strategy of electronic decoupling by an ultrathin dielectric spacer and emission enhancement by resonant plasmonic nanocavity defined by the STM junction, we demonstrate electrically driven single-photon emission from a well-defined isolated single-molecule emitter. The correlation of such single-molecule single-photon emission with the local environment also provides new insights into the exciton decay dynamics near metals at the nanometer scale. We also demonstrate the construction of single-photon emitter arrays through STM manipulation by exploiting the merit that nearly all the zinc-phthalocyanine (ZnPc) molecules on a decoupling layer can produce molecule-specific emissions.

## Results

### Single-molecule electroluminescence

**Fig. 1:**
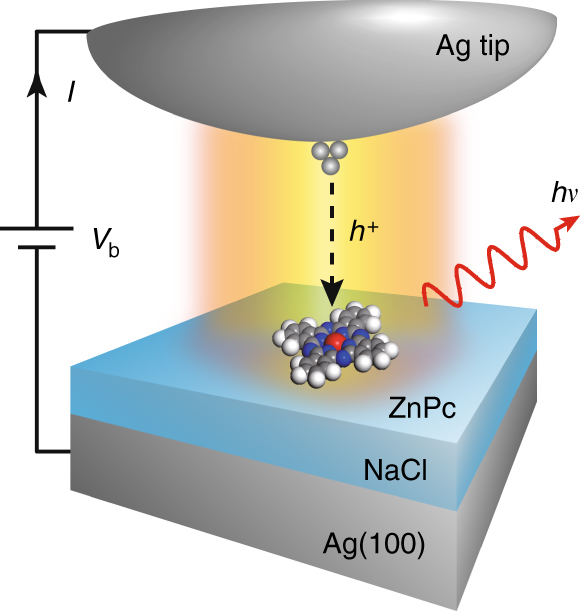
Schematic diagram of STM-induced fluorescence from a single molecule. Molecular fluorescence is generated by the excitation of highly localized tunneling electrons over a single ZnPc molecule that is decoupled by NaCl layers from the Ag(100) substrate

**Fig. 2:**
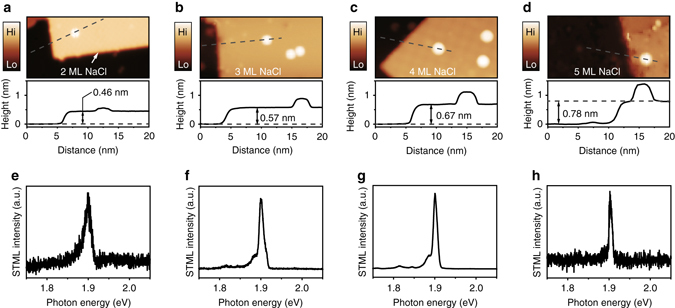
Single-molecule electroluminescence. **a**–**d** STM images of single flat-lying ZnPc molecules adsorbed on 2, 3, 4, and 5 ML thick NaCl islands, respectively (2.5 V, 2 pA, 30 × 15 nm^2^). The number of MLs is verified by apparent thicknesses measured from corresponding height profiles (along *dashed gray lines*) displayed underneath each image^40^. **e**–**h** Corresponding single-molecule electroluminescence spectra when the tip is positioned above the lobe of a ZnPc molecule on different NaCl thickness: 2 ML (**e**, −2.5 V, 200 pA, 60 s), 3 ML (**f**, −2.5 V, 100 pA, 60 s), 4 ML (**g**, −2.5 V, 100 pA, 20 s), and 5 ML (**h**, −2.5 V, 2 pA, 5 s)

Figure 1 shows the schematic of an isolated ZnPc molecular emitter electronically decoupled by a thin sodium chloride (NaCl) layer from the Ag(100) metal substrate. Silver (Ag) tips, with appropriately tuned plasmonic properties, were used to provide strong resonant plasmonic enhancement to the molecular fluorescence that is excited by highly localized tunneling electrons^33, 39^. As shown in Fig. 2, the NaCl spacer layer with a thickness of 2–5 monolayers (ML)^40^ is found to effectively suppress the fluorescence quenching and yield molecule-specific electroluminescence, as evidenced by the sharp emission peak around 1.9 eV that originates from the Q(0, 0) transition of the neutral ZnPc molecule^33, 41^. Such molecular electroluminescence could be observed for almost all isolated single molecules on the NaCl surface. Nevertheless, single-molecule electroluminescence intensities are found to vary for different NaCl thickness, revealing different quenching suppression effect. For molecules on 2 ML NaCl islands, molecular electroluminescence is still too weak to carry out photon correlation (or HBT) measurements because of relatively poor decoupling efficiency. On the other hand, for molecules on 5 ML NaCl islands with good decoupling, strong molecular electroluminescence could be obtained, but the stability for scanning over and exciting a molecule in STM becomes worse because of the thicker NaCl layer and resultant poorer conductivity. Therefore, in the following, we focus on the molecular systems adsorbed on 3 and 4 ML NaCl islands to systematically study their single-photon emission behaviors.

### Electrically driven single-molecule single-photon emission

**Fig. 3:**
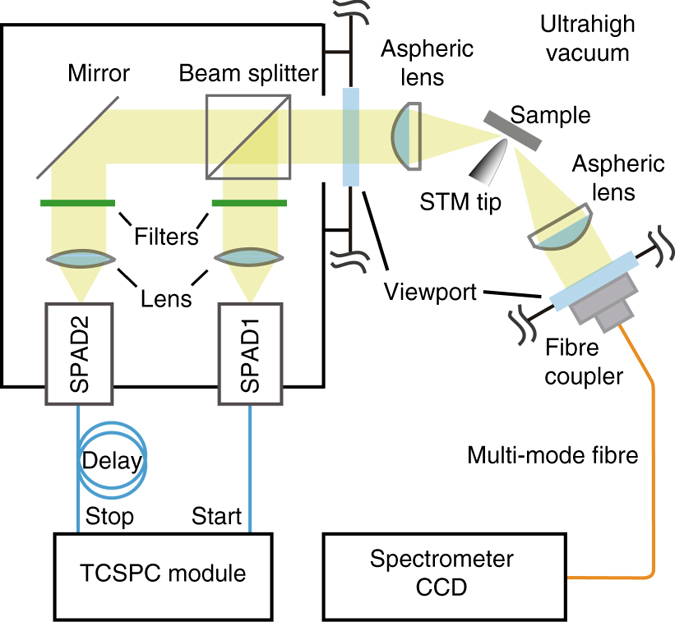
HBT setup for photon correlation measurements in STML experiments. The emitted light beam along one photon collection channel is split into two parts by a beam splitter. Two band-pass filters are set in front of two SPADs to selectively detect photons that come from the characteristic Q(0, 0) emission of the ZnPc molecule. The other photon collection channel, symmetrical to the STM tip, is connected to a CCD spectrometer for STML spectral measurements to monitor the stability of the fluorescent molecules. *TCSPC* time-correlated single-photon counting

**Fig. 4:**
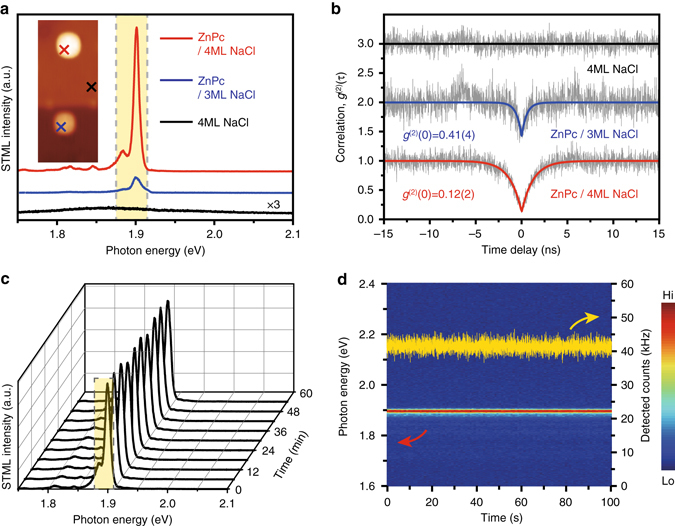
Electrically driven single-molecule single-photon emission. **a** STML spectra acquired at different sample sites using the same tip: ZnPc-lobe/4 ML NaCl, ZnPc-lobe/3 ML NaCl, and 4 ML NaCl, respectively (−2.5 V, 100 pA, 30 s). The inset of **a** shows the STM image of isolated ZnPc molecules on 3 and 4 ML NaCl (2.5 V, 2 pA, 16 × 6 nm^2^). The spectra are offset for clarity. **b** Second-order correlation measurements of single-molecule electroluminescence at the molecular lobe of a ZnPc (−2.5 V, 100 pA) and NaCl (−2.5 V, 500 pA), corresponding to the STML spectra in **a**, respectively. The *black*, *red*, and *blue lines* are the single exponential fit to the data. The *upper curves* have been upshifted by multiples of one for clarity. **c** Consecutive STML spectra acquired every 5 min during second-order correlation measurements at the ZnPc lobe on 4 ML NaCl (−2.5 V, 100 pA, 60 s). The *yellow areas* marked in **a**, **c** indicate the detection range of the band-pass filters used in the present experiments (1.872–1.915 eV). **d** Short-time trace of STML spectra (−2.5 V, 100 pA, 1 s for each spectrum) and intensities (detected by SPAD at a sampling rate of 50 Hz) when the tip is positioned at the ZnPc molecular lobe on 4 ML NaCl

A home-built HBT setup was used in the time correlation measurement of emitted photons from single ZnPc molecules excited by tunneling electrons in STM (Fig. 3). In order to make a comparable study on the photon correlation behavior of single ZnPc molecules on 3 and 4 ML NaCl islands, we carried out HBT measurements at different sample sites using the same tip (more specifically, the tip with the same plasmonic properties). Figure 4a shows two STML spectra acquired when the tip is positioned above the lobe of an isolated ZnPc molecule on 3 ML (*blue*) or 4 ML NaCl (*red*), showing about nine times stronger emission of the latter than the former. The nanocavity plasmonic emission on the bare 4 ML NaCl surface is also plotted (*black*), showing nearly resonant plasmonic mode with the molecular emission, though with a much weaker intensity. Results of photon correlation measurements for these three situations are given in Fig. 4b. An evident antibunching dip was observed in the second-order correlation function [*g* ^(2)^(*τ*)] at time zero for molecular electroluminescence from a single ZnPc molecule absorbed on either 3 or 4 ML NaCl surface. As a comparison, no dip feature was observed in the time histogram of the HBT experiment for the emission from nanocavity plasmons when the tip is positioned above the bare 4 ML NaCl surface with a certain distance from any single ZnPc molecules (e.g., >4 nm).

The observation of the antibunching dips suggests a single-photon emission behavior for the single-molecule electroluminescence. In order to evaluate the single-photon purity of the emission^13^, the experimental data of second-order correlation measurements were fitted with a single exponential function^9, 18, 42^ ${g^{\left( 2 \right)}}\left( \tau \right) = 1 - \left[ {1 - {g^{\left( 2 \right)}}\left( 0 \right)} \right]{e^{ - |\tau |/{\tau _0}}}$. The second-order correlation function at zero delay, *g* ^(2)^(0), is found to be 0.12(2) for ZnPc on 4 ML NaCl and 0.41(4) for ZnPc on 3 ML NaCl, respectively. Both *g* ^(2)^(0) values are below the threshold 0.5 and are thus a clear indication of single-photon emission^19, 25^. The corresponding time constants (*τ*_0_) are 1.26(3) ns for ZnPc on 4 ML NaCl and 0.48(5) ns for ZnPc on 3 ML NaCl, respectively, which are on the same order of magnitude as that reported for defects in C_60_ thin films (~0.69 ns)^38^, but shorter than the lifetime of ZnPc molecules in ethanol (~4.1 ns)^43^.

The single-molecule electroluminescence is found not only highly stable during the long-time HBT measurements (hours), as illustrated by the nearly identical spectra in Fig. 4c for ZnPc on 4 ML NaCl (thanks to the ultrahigh vacuum and cryogenic environment), but also free of blinking at least at the millisecond scale (Fig. 4d). The average count rate detected by a single-photon avalanche diode (SPAD) detector is ∼42 kHz at 100 pA, which corresponds to a quantum yield of ~3 × 10^−3^ photons per electron estimated by taking into account the photon collection and detection efficiency of our system (see Supplementary Note 1 for details). We would like to point out that, due to highly localized nature of tunneling electron excitations, we can ensure that in our case with isolated single molecules, only the specific molecule directly underneath the tip will be excited and produce molecular electroluminescence. Therefore, in this sense, we have successfully demonstrated strong and stable electrically driven single-photon emission from a well-defined isolated single molecule.

As shown in Fig. 4b, both the depth and width of the antibunching dip are correlated with the local environment around the single molecule. In other words, the single-photon emission behavior can be used to reveal the exciton decay dynamics of a single molecule near metals, specifically through the analysis of time constants (*τ*_0_). The reciprocal of the time constant (1/*τ*_0_) is defined as the so-called rise rate, which is generally considered to be the sum of the effective pumping rate and the decay rate of excited states (namely, excitons) in the two-level quantum system^9, 10, 24, 42^. In our experiment, the rise rate can also be understood as the sum of these two rates based on a three-state model^18, 38^ (see Supplementary Note 2 for details). For photon correlation measurements above a ZnPc molecule on 3 or 4 ML NaCl under the same current (100 pA, Fig. 4b), the pumping rate can be assumed to be constant. Therefore, the increase in the rise rate can be attributed to arise from the increase in the decay rate of the molecular excited states (i.e., the decrease in the excited-state lifetime). The significant shortening of the time constant from 1.26(3) ns for ZnPc on 4 ML NaCl spacer to 0.48(5) ns for ZnPc on 3 ML NaCl spacer corresponds to an increase in the rise rate and thus an increase in the exciton decay rate. Taking into account the dramatically reduced molecular fluorescence from ZnPc on 4 ML NaCl to ZnPc on 3 ML NaCl (Fig. 4a), such increase in the exciton decay rate can be ascribed to the rapid increase on the nonradiative decay rate of excitons to the metal substrate, resulting in shortened lifetime and stronger quenching, as expected qualitatively by the classical dipole theory^44^.

### Tip‒molecule distance dependence of single-photon emission

In the situation described above, the change in the molecular fluorescence is attributed mainly to the variations in the decoupling effect of different thickness of NaCl spacer layers from the underneath metal substrate by assuming the influence of the STM tip to be constant. However, under tunneling conditions, the metallic tip is also very close to the molecule (<1 nm, as detailed in the estimation of tip‒molecule distances in Supplementary Note 3) and will also affect the photon-emission behavior and exciton decay dynamics. Such influence can be studied by varying the tip−molecule distance for a given thickness of NaCl spacer layers, as shown below through the change of tunneling currents (as well as in Supplementary Note 4 as a function of tip‒molecule distances).

**Fig. 5:**
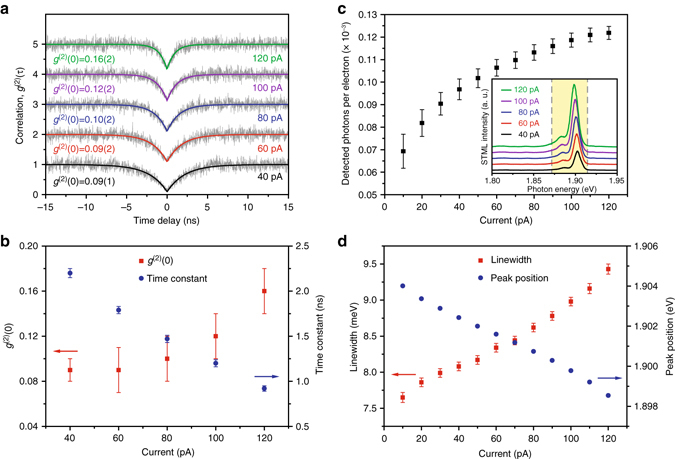
Tip–molecule distance dependence of single-photon emission. **a** Second-order correlation functions measured above the same lobe of a single ZnPc molecule on 4 ML NaCl for different tunneling currents (40–120 pA) at a given bias of −2.5 V. The *upper curves* have been upshifted by multiples of one for clarity. The start (stop) counts for the correlation measurements at 40, 60, 80, 100, and 120 pA are 24 kHz (20 kHz), 40 kHz (33 kHz), 59 kHz (48 kHz), 76 kHz (64 kHz), and 91 kHz (74 kHz), respectively, with a corresponding integration time of 9025, 3300, 1600, 1000, and 250 s. **b** Current-dependent *g* ^(2)^(0) values (*red squares*) and time constants (*blue circles*) obtained from the single exponential fit in **a**. The *error bars* represent the standard deviations obtained with the single exponential fit. **c** Emission intensities detected by one SPAD normalized by the excitation current (i.e., detected photon counts per electron) (*black squares*). The *error bars* represent the standard deviations of the detected photon counts over 900 data points (for a period of 18 s with a 50 Hz sampling rate). The inset of **c** plots corresponding STML spectra acquired simultaneously during the HBT measurements in **a** (integration time: 30 s), showing an increase in emission intensity with the increased current. The highlighted *yellow area* indicates the detection range of the band-pass filters used here (1.872–1.915 eV). **d** Red shift of peak positions and linewidth broadening for the Q(0, 0) emission along with the increase of tunneling currents. The peak positions and linewidths are obtained by the Lorentz fit to the observed STML spectra. The corresponding *error bars* represent the standard deviations obtained with the Lorentz fit

Figure 5a shows the second-order correlation functions at different currents on the same ZnPc molecule adsorbed on 4 ML NaCl. The best single-photon purity achieved in the present experiments is down to 0.09 at a tunneling current of 40 pA. When the tunneling current increases, the antibunching dips are found to be narrowed, which reflects the decrease of the corresponding time constants, as shown in Fig. 5b. This observation is consistent with the common knowledge that the total decay rate would be increased when the metallic tip approaches closer to a molecule. Notably, as illustrated in Fig. 5c, the photon-emission intensities of molecular fluorescence normalized by tunneling currents (i.e., detected photon counts per electron) exhibit an increase when the STM tip approaches closer to the molecule (no saturation in emission intensity was observed up to 120 pA). (The de-excitation mechanism by the next tunneling electron proposed in ref. ^38^ could be thus excluded in our experiments.) Since the molecule is excited through electron injection by one-electron excitation process at −2.5 V (see Supplementary Note 5 for details), the excitation efficiency of the molecule per electron is expected to be essentially the same at different tunneling currents over 40–120 pA (unless such excitation efficiency could be significantly affected by wave-function overlaps at short distances). In this case, the increase of the normalized photon intensity in Fig. 5c implies that the quantum efficiency of the molecular fluorescence is enhanced when the distance between the metallic tip and the molecule decreases. Quantum efficiency is known to be determined by the ratio of the radiative decay rate over the total decay rate. Thus, the increase of quantum efficiency suggests a stronger enhancement for the radiative decay rate over the nonradiative decay rate at smaller tip−molecule distances even less than 1 nm (see Supplementary Note 4 for details), which is different from previous reports using photoluminescence technique^45, 46^. These results may provide new facts for future theoretical modeling on molecular fluorescence enhancements for molecules in close proximity to a metallic tip and substrate.

As expected for a stronger tip−molecule interaction at smaller distances, the molecular emission peaks are found to be further red-shifted and broadened (Fig. 5d), which is also qualitatively consistent with the decrease in the time constants observed (Fig. 5b). We would like to note that the photon antibunching dip in the *g* ^(2)^(*τ*) curves acquired on single ZnPc molecules (Figs 4b, 5a) does not fall ideally to zero. This is probably due to either the presence of small background contribution from the nanocavity plasmonic emission in our optical measurements^17, 18, 21, 23^ or the temporal resolution limit of our HBT setup^10, 11, 38^, or both.

### Artificially constructed single-photon emitter array

**Fig. 6:**
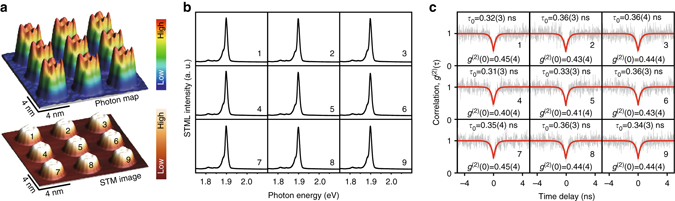
Artificially constructed single-photon emitter array. **a** Simultaneously acquired photon map (*top*) and STM image (*bottom*) of a 3 × 3 ZnPc molecular array on 3 ML NaCl through STM manipulation (−2.5 V, 50 pA, 14 × 14 nm^2^). **b**, **c** STML spectra (**b**, −2.5 V, 300 pA, 10 s) and second-order correlation functions (**c**, −2.5 V, 300 pA) acquired from corresponding ZnPc molecules marked with numbers in **a**. The start (stop) counts for the correlation measurements are around 49 kHz (50 kHz) with an integration time of 1600 s

The ability to precisely position single-photon emitters in two-dimensional space is important for the integration of on-chip quantum devices and networks^7, 8, 47^. STM is capable of manipulating single molecules with sub-nanometer precision and allows us to demonstrate an example of constructing a single-photon emitter array at nanoscale. As shown in the *bottom image* of Fig. 6a, a square 3 × 3 molecular array with intermolecular spacing of ∼4.4 nm was constructed by moving individual ZnPc molecules on 3 ML NaCl islands^33^ (see Supplementary Fig. 4 for details). The corresponding photon map measured simultaneously by a SPAD is illustrated in the *top image* of Fig. 6a, which exhibits similar emission intensity features for each individual ZnPc molecules. The spectral features, in terms of both emission peak position and intensity, are also nearly the same when acquired from the equivalent lobe positions of each individual ZnPc molecules in the array (Fig. 6b). Furthermore, as shown in Fig. 6c, the second-order correlation functions measured from each individual ZnPc molecules exhibit nearly identical antibunching features with the *g* ^(2)^(0) values all below 0.5 (around 0.40–0.45). The nearly identical feature in the spectral and single-photon emission properties arises from the facts that all the ZnPc molecules are structurally identical and behave the same on the NaCl surface, which illustrates one of the advantages to use molecular single-photon emitters in optoelectronic integration.

## Discussion

In summary, we have presented a demonstration of both electrically driven single-photon emission from a well-defined isolated single molecule and further construction of single-photon emitter arrays. Such capability not only provides new insights into the photophysics of molecular emitters near metals at the nanometer scale, but also opens up new opportunities to explore and control electrically driven single-photon sources that are important for future quantum information processing and nano-optoelectronics.

## Methods

### Sample and tip preparation

Our experiments were performed with a custom low-temperature ultrahigh-vacuum STM (Unisoku) combined with optical detection systems at about 8 K under a base pressure of about 1 × 10^−10^ Torr. ZnPc molecules were thermally evaporated onto the Ag(100) substrate partially covered by NaCl islands at about 8 K (Ag(100) was previously cleaned by argon ion sputtering and annealing). Electrochemically etched Ag tips used in all our experiments were cleaned by electron-bombarding and argon-ion sputtering. STM imaging and spectral measurements were taken in a constant-current mode with the sample biased. The manipulation of ZnPc molecules was realized via pushing following the method described in our previous work^33^.

### STML measurements

The schematic diagram of the photon collection and detection systems are shown in Fig. 3. Photons emitted from the STM junction were collected by two symmetrical channels. One collection channel is connected to a liquid-nitrogen-cooled charge-coupled device (CCD) spectrometer (Princeton Instruments) for STML spectral measurements^33^. The other channel is combined with a home-built HBT setup for the time correlation measurements of emitted photons. Photons are detected by thin-layer SPADs (Micro Photon Devices)^48^ with a 35 ps timing jitter to ensure a time resolution as good as possible. The time intervals between photons are analyzed using a time-correlated single-photon counting (TCSPC) system (PicoQuant PicoHarp 300) with 16 ps bin time. The dark count rate of the detector is below 25 Hz.

### Data availability

Data available on request from the authors.

## Electronic supplementary material

Supplementary Information
