# Supplementary material for: Electrically driven single-photon emission from an isolated single molecule
Source: Nat Commun. 2017 Sep 18;8:580. doi: 10.1038/s41467-017-00681-7 (PMC5603600; doi:10.1038/s41467-017-00681-7)
Supplement: Supplementary file 1 — Supplementary Information [file 41467_2017_681_MOESM1_ESM.pdf]

Description of Supplementary Files

File Name: Supplementary Information
Description: Supplementary Figures, Supplementary Notes and Supplementary References

Supplementary Note 1. Estimation of the total emission rate and quantum yield

The hemisphere photon collection efficiency for one optical detection channel is about 11%. The average transmittance of filters is around 95%. The detection efficiency of SPADs is about 37% around 1.90 eV. In the experiment shown in main-text Fig. 4d, the detected photon counts by two SPADs are 42 kHz and 34 kHz at an excitation current of 100 pA, respectively. Assuming an isotropic emission behaviour over the hemisphere, the total emission rate can be estimated to be ~2 MHz. Such emission rate corresponds to a quantum yield (i.e., an electron-to-photon conversion quantum efficiency) as high as $\sim 3\times10^{-3}$ photons per electron, which is of the same order of magnitude as those reported in the literatures[1,2]. The high quantum yield greatly facilitates the demonstration of single-molecule single-photon emission.

The quantum yield is improved by the adoption of a series of strategies, such as the selection of decoupling layers and emitting molecules, the adoption of silver as both tip and substrate materials, and the fine tuning of the nanocavity plasmon resonance as well as the selection of the excitation position over the molecule. The decoupling layer is mainly used to prevent fluorescence quenching due to direct electron exchange between the molecular emitter and the metal substrate. The fine tuning of the nanocavity plasmon resonance is used to provide strong plasmonic enhancement to overcome the quenching caused by the dipole-dipole energy transfer between the molecular emitter and the metal electrodes. The excitation position is selected above the lobe of the flat-lying molecule to generate strong molecular emission thanks to the symmetry breaking[3,4].

Supplementary Note 2. Three-state model for single-molecule single-photon emission

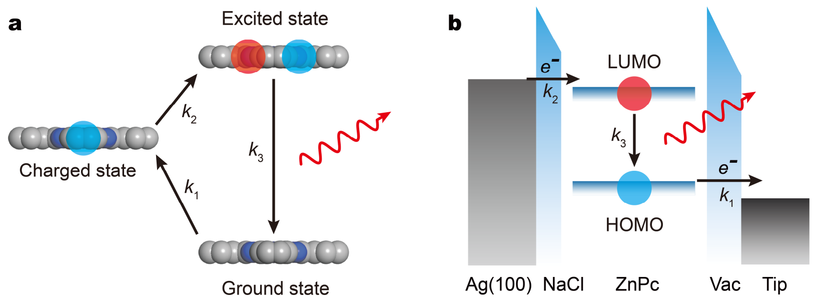

Supplementary Figure 1 | Scheme of a three-state model in STM induced molecular luminescence. a, Scheme of three states with transition rate constants k_1, k_2 and k_3. k_1 and k_2 are associated with the effective pumping rate, while k_3 corresponds to the exciton decay rate. **b,** Scheme of the STM induced molecular luminescence process in a double-barrier junction, showing the energy-level alignments of the highest occupied molecular orbitals (HOMO) and lowest unoccupied molecular orbitals (LUMO) with respect to the Fermi levels of the electrodes. The blue (red) circle in the scheme represents an electron (hole).

As shown in Supplementary Figure 1, the electrical excitation of a molecule in a double-barrier junction requires the creation of a hole (k_1) and then the capture of an electron (k_2) to generate an exciton[5,6], which is followed by the exciton decay (k_3).

Following the method used in the literature[6,7], the measured time constant τ_0 from antibunching curves in a three-state model can be expressed as:

$$\tau_0 = \left(\frac{A-\sqrt{A^2-4B}}{2} \right)^{-1} \quad (1)$$

where $A=k_1+k_2+k_3$ and $B=k_1k_2+k_2k_3+k_3k_1$.

Assuming that the electron capture process is much faster than the other two processes (i.e., $k_2 >> k_1, k_3$) as proposed in Ref. 6, Eq. (1) could be simplified as:

$$\tau_0 = \frac{1}{k_1+k_3} \quad (2)$$

The rate constant k_1 is assumed to be proportional to the tunnelling current I and can be expressed as:

$$k_1 = \alpha \frac{I}{e} \qquad (3)$$

where α is the exciton creation efficiency and e is the elementary charge.

The typical quantum yield in our experiment is estimated to be ~ 3×10^{-3} photons per electron at 100 pA for ZnPc on 4ML NaCl. Considering the fluorescence quantum yield (~ 0.3) for ZnPc molecules in photoluminescence[8], α can be estimated to be ~ 1×10^{-2}, giving rise to k_1 of ~ 0.006 ns^{-1}. Since the estimated k_1 is much smaller than the measured rise rate ($1/\tau_0$) of ~ 0.8 ns^{-1}, the contribution of k_1 to the measured rise rate can be neglected based on Eq. (2). In other word, the measured time constant τ_0 (i.e., the inverse of rise rate) can be approximated as $\tau_0 \sim 1/k_3$, which suggests that the antibunching behaviour, especially the time constant, is dominated by the molecular exciton decay process.

We would like to point out that the mechanisms of the excitation and decay process in the molecular electroluminescence here could be more complicated than the model adopted above with assumptions on the existence of an intermediate charged state and the magnitude of various rate constants. The understanding of the antibunching behaviour and associated time constants certainly deserves further theoretical and experimental studies.

Supplementary Note 3. Estimation of tip–molecule distances

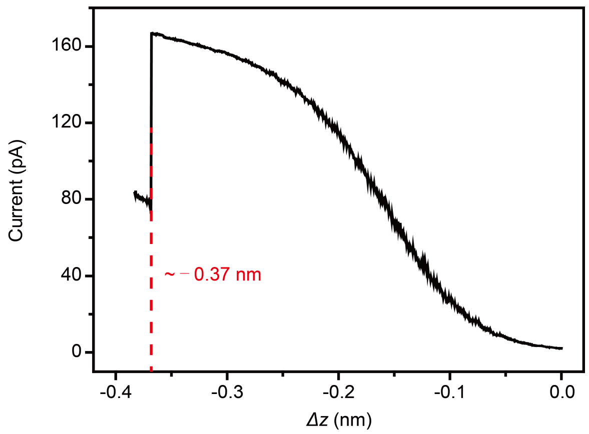

Supplementary Figure 2 | Typical *I–z* curve measured above single ZnPc molecules on 4ML NaCl. The *I–z* curve was measured with open feedback loop. The initial tip position (Δz=0) is determined by positioning the STM tip above the lobe of ZnPc molecules on 4ML NaCl at the condition: −2.5 V, 2 pA.

The *I–z* spectroscopy measurements were carried out over single ZnPc molecules on 4ML NaCl to estimate tip–molecule distances. As shown in Supplementary Figure 2, the tunnelling current monotonically increases when the tip approaches to the molecule. A sudden drop in tunnelling currents at the tip displacement (Δz) of about −0.37 nm is observed, which corresponds to the transfer of the ZnPc molecule from NaCl surface to the tip (confirmed by the missing of the target molecule in the successive STM image after the *I–z* measurement). The tip position corresponding to the sudden current drop is thus approximated as the position for the tip–molecule contact. If we further assume a distance of about 0.3 nm for such tip–molecule contact[9-11], the tip–molecule distance at different tunnelling currents could be estimated as (Δz+0.37+0.3) nm.

Supplementary Note 4. Tip–molecule distance dependence of single-photon emission

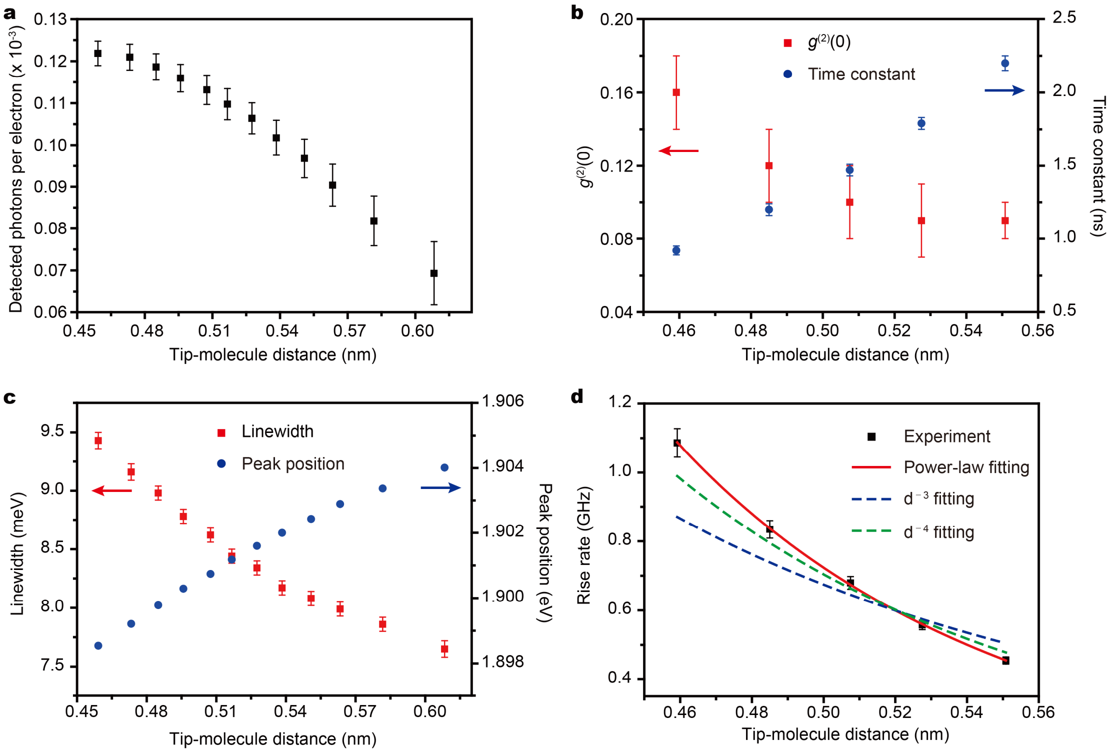

Supplementary Figure 3 | Tip–molecule distance dependence of single-photon emission. a, Emission intensities detected by one SPAD normalized by the excitation current (black squares), showing an increase with the decreased tip–molecule distances. The error bars represent the standard deviations of the detected photon counts over 900 datapoints (for a period of 18 s with a 50 Hz sampling rate). **b,** Distance-dependent $g^{(2)}(0)$ values (red squares) and time constants (blue circles) obtained from the single exponential fit in main-text Fig. 5**a**. The error bars represent the standard deviations obtained with the single exponential fit. **c,** Red shift of peak positions and linewidth broadening for the Q(0,0) emission along with the decrease of tip–molecule distances. The peak positions and linewidths are obtained by the Lorentz fitting to the observed STML spectra. The corresponding error bars represent the standard deviations obtained with the Lorentz fit. **d,** The red line is the power-law fitting of the rise rate (black squares) via the function $\tau_0^{-1} = A \cdot d^{-n}$, where A and n are fitting parameters. The dashed blue line is plotted by fitting via $\tau_0^{-1} = B \cdot d^{-3}$, where B is the fitting parameter. The dashed green line is plotted by fitting via $\tau_0^{-1} = C \cdot d^{-4}$, where C is the fitting parameter. The tip–molecule distances are estimated according to the procedure described in Supplementary Note 3 above. The error bars represent the standard deviations obtained with the single exponential fit of $g^{(2)}(\tau)$ curves.

As shown in Supplementary Figure 3, the photon yields increase and the time constants decrease at smaller tip–molecule distances. As shown in Supplementary Figure 3d, the power-law fitting via $\tau_0^{-1} = A \cdot d^{-n}$ yields an exponent of n=4.8(1), which appears deviated from the d^{-3} dependency expected for the volume damping, but somewhat closer to the d^{-4} dependency expected for the surface damping[12-14]. The deviation from the distance dependency calculated from the classical dipole-dipole energy transfer model[12-14] may be originated from the following two reasons: the difficulty in the precise definition and measurement of the tip–molecule distances and the possible failure of the point-dipole model at such small tip–molecule distances (< 1 nm). Further experimental and theoretical studies are desirable to understand the complicated distance dependency of rise rates in the present HBT experiments.

Supplementary Note 5. The exclusion of multi-electron excitation process

The multi-electron excitation process is unlikely to play a role in our experiment based on the following two considerations:

(1) As far as we know, multi-electron excitation model is usually used to explain the energy up-conversion phenomena observed in tunnelling electron induced plasmon emission[15] and molecular fluorescence[16], when the energy of a tunnelling electron is smaller than the energy of emitted photons. Nevertheless, in our experiment the excitation energy of the tunnelling electron (2.5 eV) is larger than the molecular optical bandgap and the associated photon energy of molecular fluorescence (~1.9 eV), and thus the molecule is most likely to be excited through one-electron excitation process.

(2) As shown in previous references, the photon yield per electron in multi-electron excitation process (10^{-8}~10^{-7} photons per electron for plasmon emission[15], ~1×10^{-5}

photon per electron for molecular emission[16]) is reported to be much smaller than that in one-electron excitation process (~10^{-4} photons per electron for plasmon emission[15], ~3×10^{-4} photons per electron for molecular emission[16]). The optimized photon yield per electron of molecular fluorescence reported here (~3×10^{-3} photons per electron) is much larger than the yield in the multi-electron process, but relatively close to that in the one-electron process.

Therefore, the excitation mechanism is believed to be dominated by the one-electron excitation process, rather than the multi-electron process.

Supplementary Figure 4. Construction of a 3 × 3 ZnPc molecular array through STM manipulation

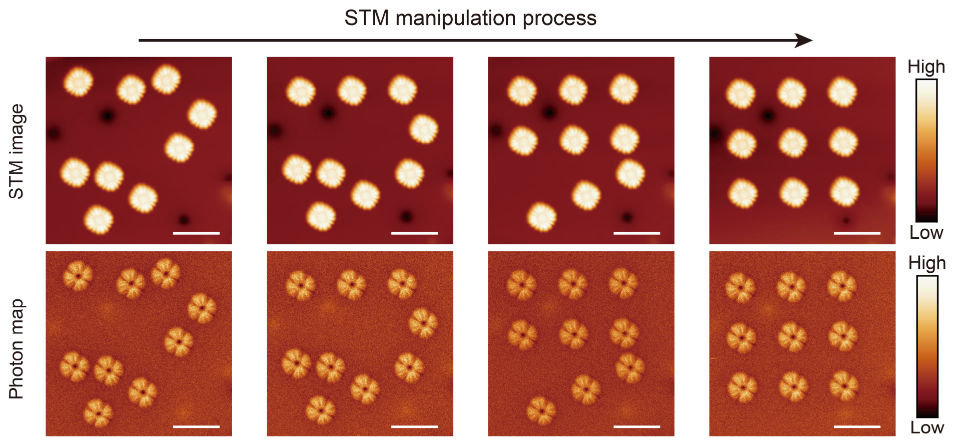

Supplementary Figure 4 | Selected images during the construction of a square 3 × 3 ZnPc molecular array by STM manipulation. Upper panel: STM images of 9 ZnPc molecules on 3ML NaCl (−2.5 V, 50 pA). Lower panel: simultaneously acquired photon maps. The scale bars in all images represent 4 nm. The manipulation of ZnPc molecules was realized via pushing following the method described in Ref. 4.

Supplementary References

1. Wu, S. W., Nazin, G. V. & Ho, W. Intramolecular photon emission from a single molecule in a scanning tunneling microscope. *Phys. Rev. B* **77,** 205430 (2008).

2. Große, C., *et al.* Submolecular electroluminescence mapping of organic semiconductors. *ACS Nano* **11,** 1230-1237 (2017).

3. Chen, C., Chu, P., Bobisch, C. A., Mills, D. L. & Ho, W. Viewing the interior of a single molecule: vibronically resolved photon imaging at submolecular resolution. *Phys. Rev. Lett.* **105,** 217402 (2010).

4. Zhang, Y., *et al.* Visualizing coherent intermolecular dipole-dipole coupling in real space. *Nature* **531,** 623-627 (2016).

5. Imamoglu, A. & Yamamoto, Y. Turnstile device for heralded single photons - Coulomb-blockade of electron and hole tunneling in quantum-confined p-i-n heterojunctions. *Phys. Rev. Lett.* **72,** 210-213 (1994).

6. Merino, P., Große, C., Roslawska, A., Kuhnke, K. & Kern, K. Exciton dynamics of C_{60}-based single-photon emitters explored by Hanbury Brown-Twiss scanning tunnelling microscopy. *Nat. Commun.* **6,** 8461 (2015).

7. Mizuochi, N., *et al.* Electrically driven single-photon source at room temperature in diamond. *Nat. Photon.* **6,** 299-303 (2012).

8. Zhang, X. F. & Xu, H. J. Influence of halogenation and aggregation on photosensitizing properties of zinc phthalocyanine (ZnPc). *J. Chem. Soc., Faraday Tran.* **89,** 3347-3351 (1993).

9. Garcia-Lastra, J. M., Rostgaard, C., Rubio, A. & Thygesen, K. S. Polarization-induced renormalization of molecular levels at metallic and semiconducting surfaces. *Phys. Rev. B* **81,** 245427 (2010).

10. Huang, Y. L., *et al.* Understanding the adsorption of CuPc and ZnPc on noble metal surfaces by combining quantum-mechanical modelling and photoelectron spectroscopy. *Molecules* **19,** 2969-2992 (2014).

11. Wagner, C., *et al.* Non-additivity of molecule-surface van der Waals potentials from force measurements. *Nat. Commun.* **5,** 5568 (2014).

12. Chance, R. R., Prock, A. & Silbey, R. Molecular fluorescence and energy transfer near interfaces. *Adv. Chem. Phys.* **37,** 1-65 (1978).

13. Persson, B. N. J. & Lang, N. D. Electron-hole-pair quenching of excited-states near a metal. *Phys. Rev. B* **26,** 5409-5415 (1982).

14. Barnes, W. L. Fluorescence near interfaces: the role of photonic mode density. *J. Mod. Opt.* **45,** 661-699 (1998).

15. Schull, G., Neel, N., Johansson, P. & Berndt, R. Electron-plasmon and

electron-electron interactions at a single atom contact. *Phys. Rev. Lett.* **102,** 057401 (2009).

16. Dong, Z. C., *et al.* Generation of molecular hot electroluminescence by resonant nanocavity plasmons. *Nat. Photon.* **4,** 50-54 (2010).
